# P-1808. Whole genome sequencing to determine mucosal selective pressure on oral poliovirus vaccine evolution

**DOI:** 10.1093/ofid/ofaf695.1977

**Published:** 2026-01-11

**Authors:** Izabela M Rezende, Frank S Zhou, Yuan J Carrington, Yvonne A Maldonado

**Affiliations:** Stanford University, Palo Alto, CA; Stanford University, Palo Alto, CA; Stanford University School of Medicine, Stanford, California; Stanford University, Palo Alto, CA

## Abstract

**Background:**

Since the Global Polio Eradication Initiative's inception in 1998, paralysis due to wild poliovirus has declined by >99%. This success is largely due to the widespread use of live attenuated Sabin oral poliovirus vaccine (OPV). However, OPV itself is unstable and long-term replication of OPV and its rapid rate of evolution can lead to genetically divergent vaccine-derived polioviruses (VDPVs). Currently, the evolution of OPV is not well characterized as most data are from samples collected during investigations triggered by acute flaccid paralysis (AFP).

**Fgiure 1.**
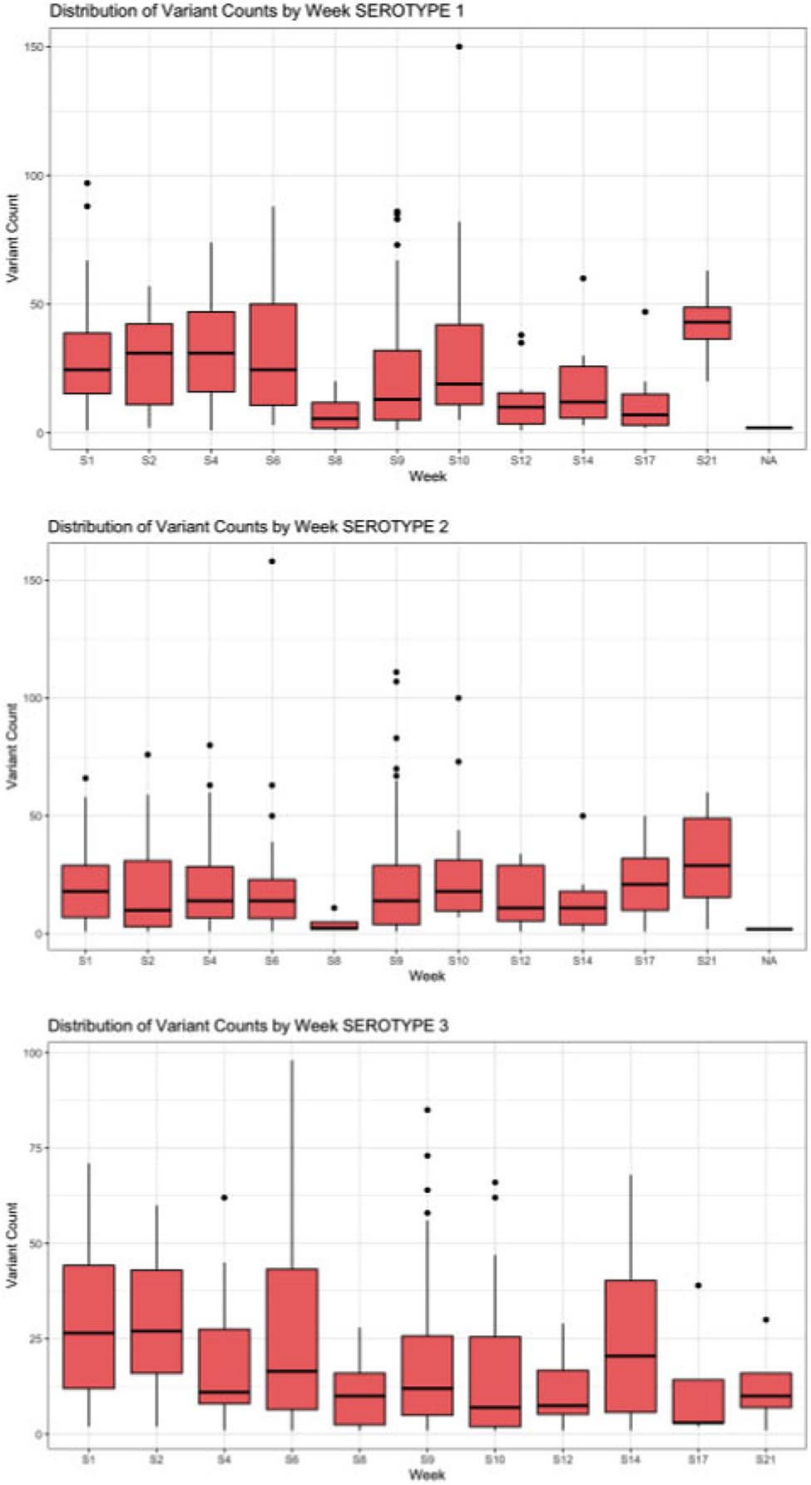
Distribution of variant count by week and poliovirus serotype. A) Serotype 1. B) Serotype 2. C) Serotype 3.

**Methods:**

Here we conducted whole genome sequencing (WGS) of 500 OPV samples collected up to eight weeks after two sequential OPV vaccine doses in Navenchauc, Mexico. The study was conducted among the entire vaccine-naïve three-year village birth cohort as they received their first two OPV doses in the 1990's. We analyzed how mucosal immunity drives OPV evolution over time, comparing WGS after dose 1 versus dose 2. Samples were sequenced using NextSeq Illumina platform. Sequencing data was processed using custom scripts based on the iVar pipeline.

**Results:**

Preliminary data showed that pre-existing immunity can influence the selection of specific OPV variants, increasing the frequency of some canonical mutations after the 2^nd^ OPV dose. We also found a significant difference in the number of non-synonymous mutations (p < 0.005) in samples collected from weeks 1- 8 (first dose of OPV) compared to weeks 9 - 17 (after 2nd dose of OPV).

**Conclusion:**

Children who have been previously vaccinated may exert different selective pressures on the virus compared to those who are immunologically naïve. We must understand these complex interactions between host immunity and viral evolution. These are crucial to predict emergence of VDPVs, develop relevant routine immunization programs, and inform strategies to prevent future OPV and VDPV outbreaks.

**Disclosures:**

Yvonne A. Maldonado, MD, Pfizer (Any division): Grant/Research Support|Pfizer (Any division): Past member, DSMB

